# Characterization of [^11^C]Lu AE92686 as a PET radioligand for phosphodiesterase 10A in the nonhuman primate brain

**DOI:** 10.1007/s00259-016-3544-9

**Published:** 2016-11-05

**Authors:** Kai-Chun Yang, Vladimir Stepanov, Nahid Amini, Stefan Martinsson, Akihiro Takano, Jacob Nielsen, Christoffer Bundgaard, Benny Bang-Andersen, Sarah Grimwood, Christer Halldin, Lars Farde, Sjoerd J. Finnema

**Affiliations:** 1 Department of Clinical Neuroscience, Center for Psychiatric Research, Karolinska Institutet, Karolinska University Hospital, Stockholm, Sweden; 2 Synaptic Transmission, H. Lundbeck A/S, Valby, Denmark; 3 Discovery Chemistry and DMPK, H. Lundbeck A/S, Valby, Denmark; 4 Neuroscience and Pain Research Unit, Pfizer Inc., Cambridge, MA USA; 5 Personalized Health Care and Biomarkers, AstraZeneca PET Science Center at Karolinska Institutet, Stockholm, Sweden; 6 Present Address: Department of Radiology and Biomedical Imaging, Yale University, New Haven, CT USA

**Keywords:** [^11^C]Lu AE92686, Monkey, MP-10, Phosphodiesterase 10A, PET, Substantia nigra

## Abstract

**Purpose:**

[^11^C]Lu AE92686 is a positron emission tomography (PET) radioligand that has recently been validated for examining phosphodiesterase 10A (PDE10A) in the human striatum. [^11^C]Lu AE92686 has high affinity for PDE10A (*IC*_50_ = 0.39 nM) and may also be suitable for examination of the substantia nigra, a region with low density of PDE10A. Here, we report characterization of regional [^11^C]Lu AE92686 binding to PDE10A in the nonhuman primate (NHP) brain.

**Methods:**

A total of 11 PET measurements, seven baseline and four following pretreatment with unlabeled Lu AE92686 or the structurally unrelated PDE10A inhibitor MP-10, were performed in five NHPs using a high resolution research tomograph (HRRT). [^11^C]Lu AE92686 binding was quantified using a radiometabolite-corrected arterial input function and compartmental and graphical modeling approaches.

**Results:**

Regional time-activity curves were best described with the two-tissue compartment model (2TCM). However, the distribution volume (*V*_T_) values for all regions were obtained by the Logan plot analysis, as reliable cerebellar *V*_T_ values could not be derived by the 2TCM. For cerebellum, a proposed reference region, *V*_T_ values increased by ∼30 % with increasing PET measurement duration from 63 to 123 min, while *V*_T_ values in target regions remained stable. Both pretreatment drugs significantly decreased [^11^C]Lu AE92686 binding in target regions, while no significant effect on cerebellum was observed. Binding potential (*BP*_ND_) values, derived with the simplified reference tissue model (SRTM), were 13–17 in putamen and 3–5 in substantia nigra and correlated well to values from the Logan plot analysis.

**Conclusions:**

The method proposed for quantification of [^11^C]Lu AE92686 binding in applied studies in NHP is based on 63 min PET data and SRTM with cerebellum as a reference region. The study supports that [^11^C]Lu AE92686 can be used for PET examinations of PDE10A binding also in substantia nigra.

**Electronic supplementary material:**

The online version of this article (doi:10.1007/s00259-016-3544-9) contains supplementary material, which is available to authorized users.

## Introduction

Phosphodiesterase 10A (PDE10A) is a member of the cyclic nucleotide phosphodiesterase family and modulates intracellular signal transduction by catabolizing cyclic adenosine monophosphate (cAMP) and guanosine monophosphate (cGMP) [1]. The intracellular enzyme PDE10A is almost exclusively expressed in GABAergic medium spiny neurons of the striatum, where it is distributed to both cell bodies and processes at presynaptic sites within globus pallidus (GP) and substantia nigra (SN) [1–5]. The localization in the basal ganglia and interaction with dopaminergic neurotransmission [5, 6] suggest that PDE10A may play a role in striatal functions such as cognitive and motor abilities [1, 6, 7]. PDE10A has, therefore, been considered as a potential therapeutic target for several neuropsychiatric disorders, including schizophrenia, bipolar disorder, Huntington’s disease and Parkinson’s disease [1, 7, 8].

Numerous potential radioligands for imaging of PDE10A binding by positron emission tomography (PET) have been suggested and several have been examined in nonhuman primates (NHP) or in humans [8–19]. Most of these radioligands provide adequate target-to-background signal for the striatum and GP, but so far, radioligand binding in the SN has been examined only for [^18^F]JNJ-42259152 or [^11^C]IMA107. Both studies report binding potential (*BP*_ND_) values of approximately 0.5 for SN in healthy human subjects with corresponding striatal *BP*_ND_ values of 3.5 or 2.2, respectively [8, 16]. This low *BP*_ND_ value might limit the application of these radioligands for quantitative examinations of PDE10A binding in SN [20]. Considering that the SN is a key nucleus in relation to the basal ganglia [21], and that regional differences in PDE10A functioning may be a part of the pathophysiology of neuropsychiatric disorders [2, 5, 6], it is important to develop a PET methodology enabling quantification of PDE10A binding in the SN of the primate brain.

[^11^C]Lu AE92686 is a recently developed radioligand with high affinity for PDE10A (*IC*_50_ = 0.39 nM). An evaluation of the radioligand in humans has previously been reported [9]. The characteristics of this radioligand are promising, with striatal *BP*_ND_ value around 7.5 in humans [9], which is higher than those reported for other PDE10A radioligands (typical range: 2–5) [8–19]. Preliminary examination of [^11^C]Lu AE92686 in cynomolgus monkeys has suggested that [^11^C]Lu AE92686 binding is high in the striatum (*BP*_ND_ around 6.5), and the specificity of binding was demonstrated by dose-dependent reduction in uptake of [^11^C]Lu AE92686 following the administration of PDE10A inhibitor MP-10 [9]. However, the initial study in monkeys did not include a full kinetic evaluation of [^11^C]Lu AE92686 or examination of binding in the SN.

The aim of the present study was to further characterize the binding properties of [^11^C]Lu AE92686 in NHPs. To enable examinations of PDE10A binding in the SN, PET measurements were conducted using the High Resolution Research Tomograph (HRRT). For full kinetic quantification of [^11^C]Lu AE92686 binding, a metabolite-corrected arterial input function (AIF) was obtained. Moreover, pretreatment experiments with unlabeled Lu AE92686 or the structurally unrelated PDE10A inhibitor MP-10 were performed to further characterize the specificity of binding and to evaluate the suitability of cerebellum as a reference region.

## Materials and methods

### Subjects

The study was approved by the Animal Research Ethical Committee of the Northern Stockholm region (Dnr N452/11, N632/12, N633/12 and N185/14). Five female cynomolgus monkeys (*Macaca fascicularis*) with body weight: 6.4 ± 1.7 kg (mean±standard deviation (SD)), for all following values with the same format) were included. The caring and experimental procedures were performed according to the ‘Guidelines for planning, conducting and documenting experimental research’ (Dnr 4820/06-600) of Karolinska Institutet and the ‘Guide for the Care and Use of Laboratory Animals: Eighth Edition’ [22].

### Preparation of [^11^C]Lu AE92686

[^11^C]Lu AE92686 was prepared according to procedures reported previously [9] as described in Online Resource 1.

### Study design

A total of 11 PET measurements with arterial blood sampling were performed on seven experimental days. Each monkey underwent one baseline PET measurement on each experimental day and on four experimental days a consecutive PET measurement was conducted after pretreatment with MP-10 (1.5 mg/kg, NHP1 and NHP2) or Lu AE92686 (0.5 mg/kg or 2.0 mg/kg, NHP1). The interval between the two PET measurements on the same day was approximately 3 h.

### Test drug administration

MP-10 [23] was formulated in a mixture of PEG 400 (25 %, v/v) and 0.9 % saline (75 %, v/v), and Lu AE92686 was formulated in a mixture of 10 % hydroxypropyl beta cyclodextrin dissolved in phosphate buffered saline (PBS). All drug solutions were infused (∼1 mL/kg) over 15 min, starting 45 min before injection of [^11^C]Lu AE92686.

### PET experimental procedures

Anesthesia was initiated by intramuscular injection of ketamine hydrochloride (∼10 mg/kg) and maintained by sevoflurane (2–8 %). PET measurements were conducted in the HRRT; a six-minute transmission scan (using a single Cesium-137 source) was followed by the acquisition of list-mode data for 123 min after a bolus injection of [^11^C]Lu AE92686.

Arterial blood samples were collected continuously during the first 3 min after radioligand injection by using an automated blood sampling system, and subsequent arterial blood samples were taken manually. Manual samples included one sample for determination of protein binding (5 min before radioligand injection), 14 samples (at 0.5, 1.0, 1.5, 2.0, 2.5, 3.0, 4.0, 5.0, 8.0, 15, 30, 60, 90 and 120 min) for measurement of blood and plasma radioactivity and seven samples (at 2.5, 4.0, 15, 30, 60, 90 and 120 min) for radiometabolite analyses. In the pretreatment experiments, five additional blood samples (at 15, 30, 60, 90 and 120 min) were taken for determination of plasma drug concentrations.

### Blood samples analysis

The free fraction (*f*_p_) of [^11^C]Lu AE92686 in plasma was estimated by ultrafiltration, and the percentages of radioactivity for unchanged radioligand and radiometabolites in plasma were determined by reversed-phase high-performance liquid chromatography (HPLC) according to procedures reported previously [14, 24]. Plasma drug concentrations were determined using ultra performance liquid chromatography (UPLC) followed by tandem mass spectrometry (MS/MS) detection according to procedures reported previously [25]. The details of the blood sample analysis are described in Online Resource 1.

### Image data analysis

Each monkey underwent magnetic resonance imaging (MRI), and the images were used for anatomical guidance to define regional volumes of interest (VOIs). PET images were preprocessed according to previously reported methods [26] with reconstructed image frames binned as: 9 × 20 s, 3 × 60 s, 5 × 180 s and 17 × 360 s. Each subject’s baseline blood flow by dominated PET image (average of time frames corresponding to 0–9 min) was coregistered manually to their individual MRI image, and the resulting transformation matrices were applied to the corresponding dynamic PET image. For pretreatment studies, the transformation parameters of the baseline measurements were applied to both of the two PET measurements performed on the same day.

Six VOIs were defined, including three striatal regions: putamen, caudate nucleus (CN) and ventral striatum (VS), two extrastriatal regions: GP and SN and one reference region: cerebellum. A decay-corrected time–activity curve (TAC) was generated for each VOI from the coregistrated dynamic PET data and radioactivity was expressed as standardized uptake values (SUV), which were calculated from the radioactivity concentration as [kBq/cm^3^] / (radioactivity injected [MBq] / body weight [kg]). Specific binding was defined as radioactivity in target region minus radioactivity in cerebellum.

### Quantification of PET signals

Kinetic analysis was performed by applying the 1-tissue compartment model (1TCM) and the 2-tissue compartment model (2TCM). Logan plot analysis [27] was also applied and the starting time of linearization (t*) was decided for each VOI in each experiment separately by examining total distribution volume (*V*_T_) and identifiability of *V*_T_ based on different t* ranging from 6 min to a time point with five time frames remaining until the end of PET measurement. In addition to the acquisition of 123 min data, the effects of the PET measurement duration on *V*_T_ estimates were examined by varying the time interval from 0 to 33 min to 0–117 min with one time frame increments.* BP*_ND_ was derived by three approaches, the indirect method using the following equation

1 $$ B{P}_{ND}=\frac{\mathrm{Regional}\kern0.5em {V}_T-\mathrm{Cerebellar}\kern0.5em {V}_T}{\mathrm{Cerebellar}\kern0.5em {V}_T}, $$

and two reference tissue models: the simplified reference tissue model (SRTM) [28] and Logan reference tissue model (Loganref) [29]. The efflux rate constant *k*_2_′ in Loganref was derived by using SRTM and couple fitting of all target regions except SN (excluded due to its relatively high noise levels). The t* for Loganref was 27 min, based on the results of the Logan plot analysis.

All analyses of imaging data were performed using PMOD (version 3.403; PMOD Technologies, Zurich, Switzerland). The fits obtained by different models were evaluated by the model selection criterion (MSC) [30], for which a higher value indicates a better fit. The identifiability of parameters was expressed by the percentage of the coefficient of variation (%COV) calculated according to the following equation

2 $$ \%\mathrm{C}\mathrm{O}\mathrm{V}=\left(\mathrm{Standard}\kern0.9mm \mathrm{error}\kern0.9mm \mathrm{of}\kern0.9mm \mathrm{estimates}/\mathrm{Estimates}\kern0.9mm \mathrm{of}\kern0.9mm \mathrm{parameters}\right)\times 100. $$

Poor identifiability was defined as %COV > 10 %. For the pretreatment studies, Lassen plots [31] were applied to estimate the nondisplaceable distribution volume (*V*_ND_) and PDE10A occupancy. Due to the significant difference of the plasma free fraction (*f*_p_) before and after pretreatment (see results section), the evaluation of pretreatment effect was based on *V*_T_ or *V*_ND_ values normalized for *f*_p_ as *V*_T_/*f*_p_ or *V*_ND_/*f*_p_, respectively.

## Results

All PET measurements were performed according to the study protocol and no significant adverse effects were observed during the pretreatment experiments.

## Radiochemistry

The radiochemical purity of [^11^C]Lu AE92686 was higher than 99 %, and the average specific radioactivity at time of injection was 1062 GBq/μmol (range: 609–1457 GBq/μmol), corresponding to a mean injected mass of 0.07 μg (range: 0.04–0.12 μg).

## Plasma radioactivity and radiometabolite analysis

**Fig. 1:**
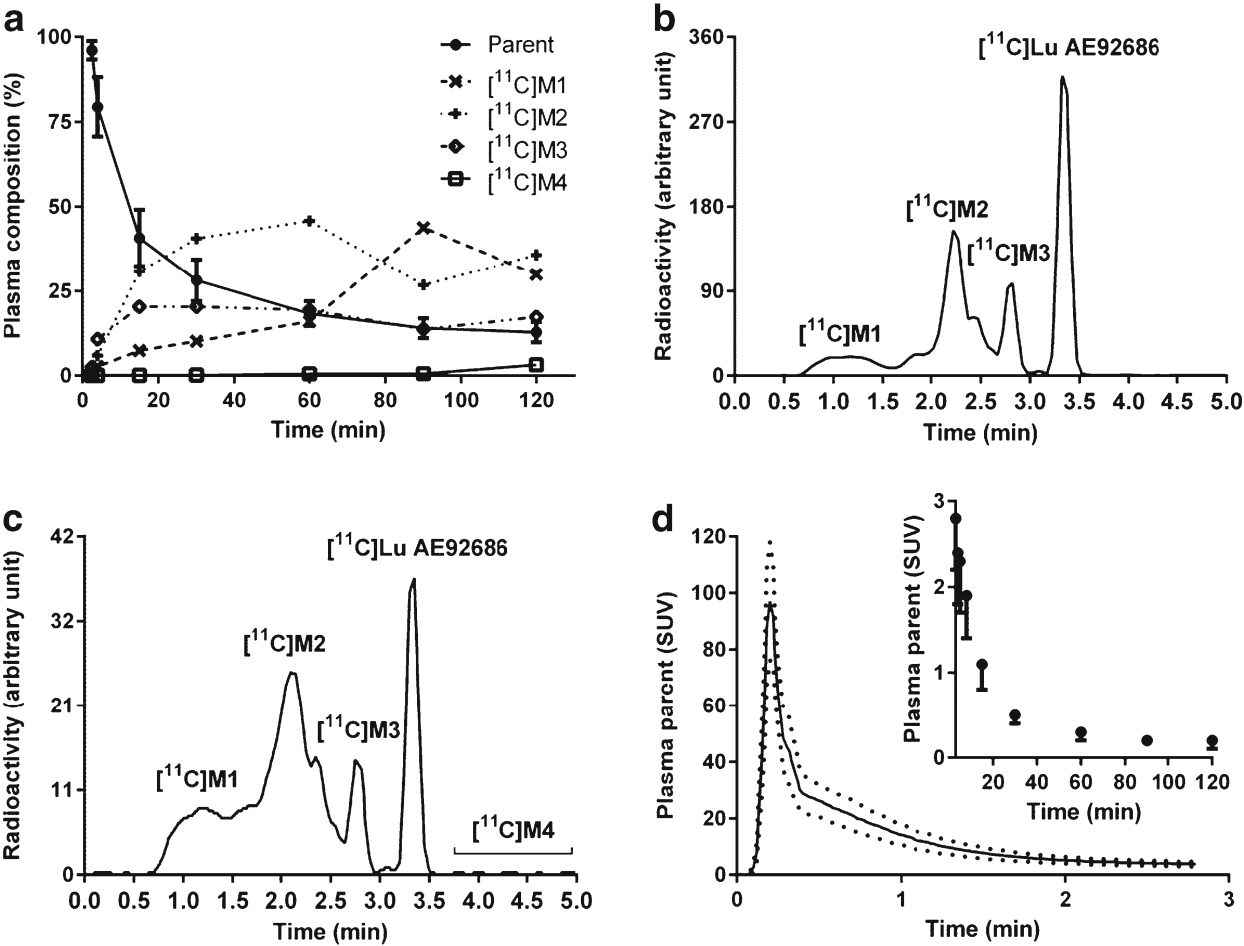
Composition of radioactivity in arterial plasma samples after [^11^C]Lu AE92686 injection in baseline experiments. (**a**) Plasma composition of unchanged radioligand (mean±SD) and radiometabolites (mean) fractions over time (*n* = 7) (**b**) Representative HPLC radiochromatogram of plasma content 15 min post injection (**c**) HPLC: 60 min post injection (**d**) Radiometabolite-corrected arterial input function of [^11^C]Lu AE92686 (*n* = 7) from 0 to 3 min (mean±SD); inset shows values from 3 to 120 min (mean – SD), expressed in standardized uptake value (SUV)

[^11^C]Lu AE92686 was rapidly metabolized and parent compound fraction was 18 ± 4 % of the plasma radioactivity at 60 min after injection (Fig. 1a). At 15 min after injection, three main radiometabolite fractions ([^11^C]M1 – [^11^C]M3) eluted earlier than [^11^C]Lu AE92686 (Fig 1b). At later time points, [^11^C]M1 and [^11^C]M2 could not be separated and the combined fraction increased with time until 90 min post injection (Fig. 1a). In addition, several small radiometabolite fractions ([^11^C]M4) eluted later than [^11^C]Lu AE92686 (Fig. 1c). The time course of plasma radioactivity of parent [^11^C]Lu AE92686 at baseline measurements (*n* = 7) is presented in Fig. 1d.

## Quantification of [^11^C]Lu AE92686 receptor binding

**Fig. 2:**
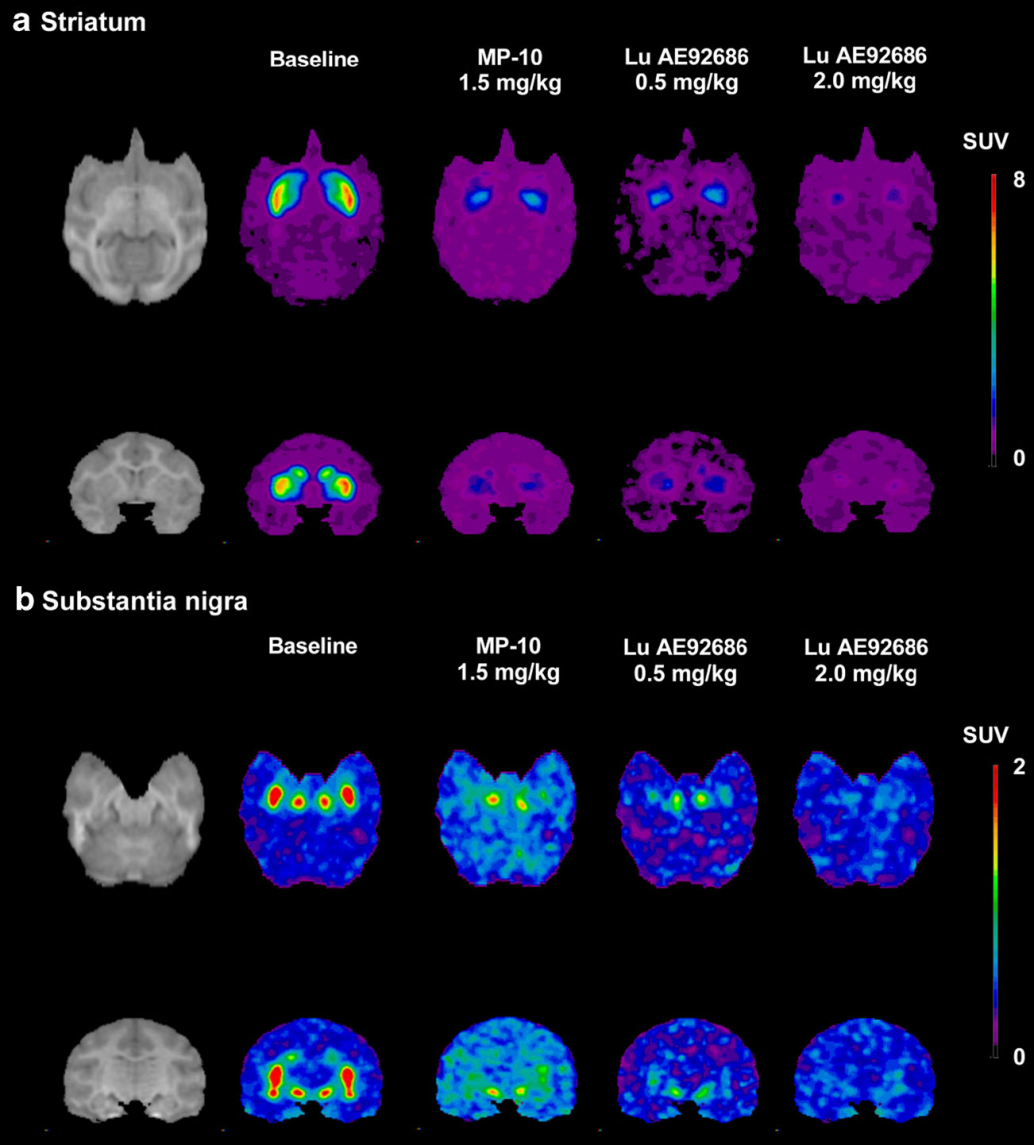
Magnetic resonance images and corresponding coregistrated PET summation images (average of frames from 9 to 123 min) of [^11^C]Lu AE92686 during baseline (mean of three baseline experiments) and three pretreatment conditions in NHP1. (**a**) Axial (top) and coronal (bottom) view of images at the level of striatum (**b**) Axial (top) and coronal (bottom) view of images at the level of substantia nigra; note the difference in the range of the standardized uptake value (SUV) colour bars between (**a**) and (**b**)

**Fig. 3:**
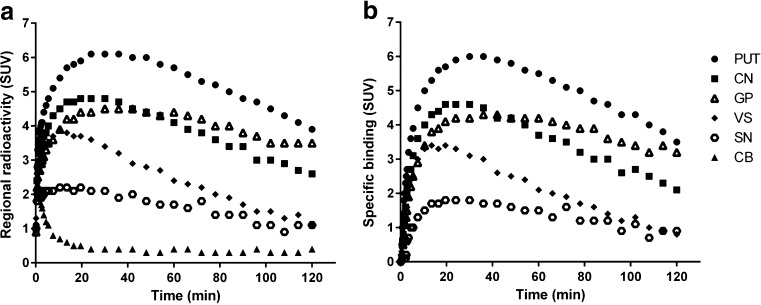
Time-activity course of radioactivity in brain for baseline experiments (*n* = 7), expressed in standardized uptake value (SUV). (**a**) Mean regional brain radioactivity concentrations in five target regions: putamen (PUT), caudate nucleus (CN), globus pallidum (GP), ventral striatum (VS), and substantia nigra (SN) as well as one reference region: cerebellum (CB) (**b**) Mean regional specific binding (radioactivity in target region - radioactivity in cerebellum) in five target regions

After intravenous injection of [^11^C]Lu AE92686 at baseline conditions, there was a rapid increase of radioactivity in regions known to express PDE10A (Fig. 2, left column). The TACs for target regions and cerebellum are shown in Fig. 3a. Specific binding, when defined as the difference between radioactivity in target region and the cerebellum, reached maximum values within 60 min after injection for all target regions (Fig. 3b).

**Fig. 4:**
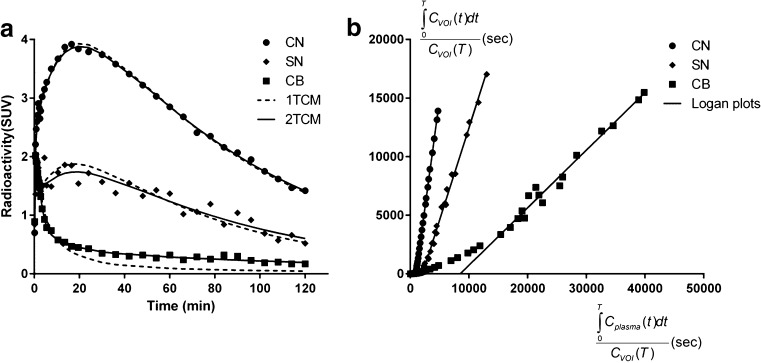
Representative kinetic modelling evaluation of [^11^C]Lu AE92686 in one NHP. (**a**) One-tissue compartment model (1TCM) fits and two-tissue compartment model (2TCM) fits in the caudate nucleus (as in putamen and globus pallidus), substantia nigra (as in ventral striatum), and cerebellum (**b**) Corresponding Logan plot analysis described the data adequately for all regions

The regional TACs were first interpreted by the 1TCM and 2TCM. For target regions, both models appeared to describe experimental data equally well. For cerebellum, the 2TCM described TACs better than the 1TCM (Fig. 4a). A summary of model parameters is given in Table 1. There were statistically higher MSC values for the 2TCM than for 1TCM in SN (*P* < 0.01) and in cerebellum (*P* < 0.0001) as well as when pooling all six regions (2.8 ± 1.3 and 3.1 ± 1.3, *P* < 0.0001). For the 2TCM, mean *K*_1_ values ranged from 0.06 to 0.11 with mean %COV from 3 to 17 %. The estimated *k*_3_, *k*_4_, *K*_1_/*k*_2_ and *k*_3_/*k*_4_ values suffered from poor identifiability (%COV > 50 % in most fits). Furthermore, the 2TCM had poor *V*_T_ identifiability (%COV > 10 %) in all fits for the cerebellum and in a few fits for the target regions (four of 35 fits).

**Table 1:** Parameters estimated by the 1-tissue compartment model (1TCM) and the 2-tissue compartment model (2TCM) in baseline PET measurements (*n* = 7)

	1TCM		2TCM				
	*V*_T_ (ml · cm^−3^)	MSC	*V*_T_ (ml · cm^−3^)	MSC	*K*_1_^a^	*k*_3_ (min^−1^)	*k*_4_ (min^−1^)
	%COV		%COV		%COV	%COV	%COV
PUT	11.9 ± 3.8	4.2 ± 0.7	11.8 ± 3.7	4.3 ± 0.5	0.11 ± 0.02	0.55 ± 0.36	0.158 ± 0.105
	2 ± 1		2 ± 1		6 ± 5	61 ± 43	79 ± 67^b^
CN	7.5 ± 2.4	3.8 ± 0.9	7.4 ± 2.4	4.0 ± 0.6	0.10 ± 0.02	0.34 ± 0.21^d^	0.137 ± 0.094^d^
	2 ± 1		4 ± 6		4 ± 2	69 ± 52^d^	48 ± 47^d^
GP	10.3 ± 3.1	2.9 ± 0.7	10.3 ± 3.1	3.0 ± 0.5	0.08 ± 0.02	0.49 ± 0.48	0.058 ± 0.024^b^
	4 ± 1		5 ± 2		17 ± 13	124 ± 8	113 ± 34^b^
VS	3.8 ± 1.2	2.2 ± 0.4	4.0 ± 1.3^b^	2.3 ± 0.2	0.10 ± 0.03	0.44 ± 0.63	0.080 ± 0.036^b^
	3 ± 1		4 ± 2^b^		9 ± 6	124 ± 128	63 ± 35^b^
SN	2.7 ± 0.9	0.8 ± 0.6	2.6 ± 0.7^b^	1.1 ± 0.5*	0.06 ± 0.01^b^	0.26 ± 0.33	0.038 ± 0.009^b^
	7 ± 3		7 ± 2^b^		11 ± 4^b^	82 ± 29	38 ± 23^b^
CB	0.2 ± 0.1	2.8 ± 0.5	0.9 ± 0.4^e^	4.2 ± 0.6*	0.09 ± 0.02	0.02 ± 0.01	0.003 ± 0.004^c^
	6 ± 2		28 ± 16^e^		3 ± 1	23 ± 6	144 ± 130^c^

Data presented as mean±SD

*V*_*T*_ total distribution volume, *COV* the coefficient of variation, *MSC* model selection criterion, *PUT* Putamen, *CN* Caudate nucleus, *GP* Globus pallidum, *VS* Ventral striatum, *SN* Substantia nigra, *CB* Cerebellum

^a^unit = ml · min^−1^ · cm^−3^

^b^ *n* = 6; ^c^ *n* = 5; ^d^ *n* = 4; ^e^ *n* = 3 (excluding data with %COV > 50 % for *V*_T_ or *K*_1_ and data with %COV > 500 % for *k*_3_ or *k*_4_)

*P < 0.05 (two tailed, comparing the results for 1TCM and 2TCM by paired *t*-test)

Data were also interpreted by Logan plot analysis with AIF (Fig. 4b), and identifiable *V*_T_ estimates (%COV ≤ 10 %) could be derived for all regions and all subjects. *V*_T_ values by Logan plot analysis correlated with those obtained by the 2TCM with %COV ≤ 10 % (Pearson *r* > 0.99, *P* < 0.0001, *n* = 30). However, the values were slightly lower than those obtained using 2TCM (−6.3 ± 2.6 %). When comparing the two models, the equation for the linear regression analysis was y = 0.934x + 0.015 (R^2^ > 0.99). In the following, the analyses were based on the *V*_T_ values obtained by Logan plot analysis, since this model provided well identifiable values for all regions, including cerebellum.

**Fig. 5:**
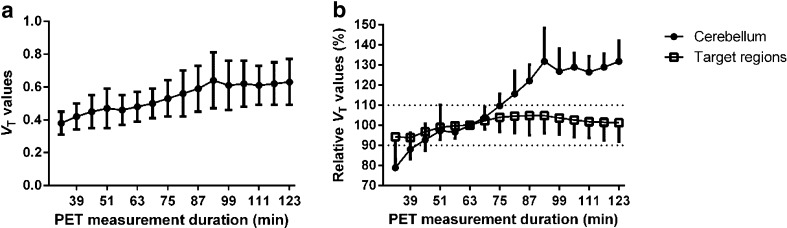
Effect of PET measurement duration on *V*_T_ values derived by the Logan plot analysis; Relative *V*_T_ values (%) = [(*V*_T_ values by corresponding duration of PET measurement - *V*_T_ values by reference duration of PET measurement) / *V*_T_ values by reference duration of PET measurement] × 100 (**a**) Cerebellar *V*_T_ values (mean±SD) (**b**) Relative *V*_T_ values to 63 min data for target regions (mean – SD) and cerebellum (mean+SD)

In target regions and PET data set of less than 63 min, *V*_T_ estimates were unstable (high variation with different t*) and poorly identifiable *V*_T_ estimates were obtained for several regions (13 of 35 fits for 33 min data). The *V*_T_ values for the cerebellum increased prominently with longer PET measurement duration as *V*_T_ values for 33 min, 93 min and 123 min data accounted for 78.9 ± 3.4 %, 131.8 ± 16.6 % and 131.8 ± 10.4 % of the *V*_T_ obtained for 63 min data, respectively (Fig. 5a and b). The *V*_T_ values of target regions remained stable after increasing PET measurement duration, and *V*_T_ values for 123 min data accounted for 101.3 ± 9.3 % of the *V*_T_ obtained for 63 min data (Fig. 5b). To minimize the possible influence of radiometabolites (see discussion section), 63 min ﻿was chosen as the preferred PET measurement duration.

## Pretreatment experiments

Five blood samples were obtained within the period of the PET measurements for assessment of the plasma concentration of the pretreatment drugs. The mean plasma concentrations were 685 and 749 ng/mL after injection of MP-10, 109 ng/mL after injection of Lu AE92686 0.5 mg/kg and 388 ng/mL after Lu AE92686 2.0 mg/kg. (Fig. S1 in Online Resource 2). The metabolic rate of [^11^C]Lu AE92686 after pretreatment was similar to baseline conditions (Fig. S2 in Online Resource 2).

**Fig. 6:**
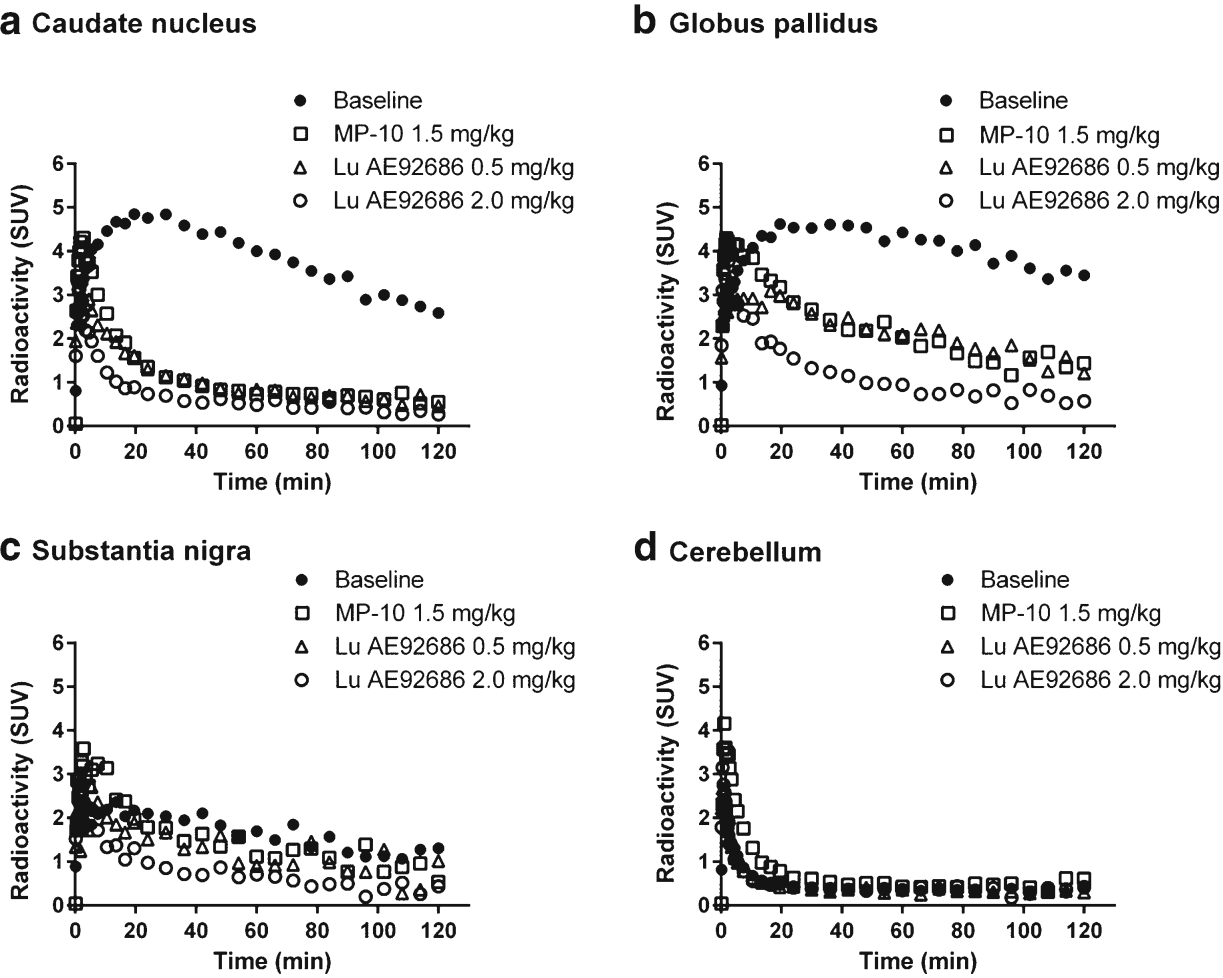
Time-activity course for radioactivity in brain for baseline experiments (mean of three measurements) and corresponding pretreatment measurements of [^11^C]Lu AE92686 in one NHP, expressed in standardized uptake value (SUV). Regional brain radioactivity concentrations in (**a**) caudate nucleus, (**b**) globus pallidus, (**c**) substantia nigra and (**d**) cerebellum

Following administration of MP-10 or Lu AE92686, radioactivity was lower in all target regions when compared to baseline (Fig. 6a–c). Radioactivity concentrations in the cerebellum were similar between baseline and pretreatment conditions (Fig. 6d). The pooled *K*_1_ values obtained by the 2TCM were 0.092 ± 0.029 at baseline conditions and 0.221 ± 0.114 (*P* < 0.0001) after pretreatment, suggesting a drug effect on blood flow.

The plasma free fraction (*f*_p_) of [^11^C]Lu AE92686 was 0.06 ± 0.01 during baseline conditions, whereas it was higher after administration of MP-10 (0.14 and 0.09) or Lu AE92686 (0.07 and 0.15 for 0.5 mg/kg and 2.0 mg/kg, respectively). Both MP-10 and Lu AE92686 induced a significant reduction in *V*_T_/*f*_p_ in target regions, and the reduction in *V*_T_/*f*_p_ was larger after the higher dose of Lu AE92686 (Table 2). For the cerebellum, there was no statistical significant difference (4.8 ± 15.4 %, *P* = 0.55) in *V*_T_/*f*_p_ between baseline (8.6 ± 2.5) and pretreatment (9.0 ± 2.5) conditions (Table 2).

**Table 2:** Changes in regional *V*_T_ / *f*_p_ (%)^a^ following three different pretreatment regimens

	MP-10 1.5 mg/kg	MP-10 1.5 mg/kg	Lu AE92686 0.5 mg/kg	Lu AE92686 2.0 mg/kg
PUT	−89.2	−87.9	−80.5	−93.7
CN	−88.0	−86.3	−76.4	−92.8
GP	−76.4	−76.2	−54.9	−90.3
VS	−81.6	−77.2	−66.2	−89.8
SN	−46.7	−43.2	−23.4	−75.8
CB	1.0	−3.5	26.3	−9.7

*PUT* Putamen, *CN* Caudate nucleus, *GP* Globus pallidum, *VS* Ventral striatum, *SN* Substantia nigra, *CB* Cerebellum

^a^Change in *V*_T_ / *f*_p_ (%) = ($$ \frac{\mathrm{Pretreatment}-\mathrm{Baseline}}{\mathrm{Baseline}}\Big)\times 100 $$

**Fig. 7:**
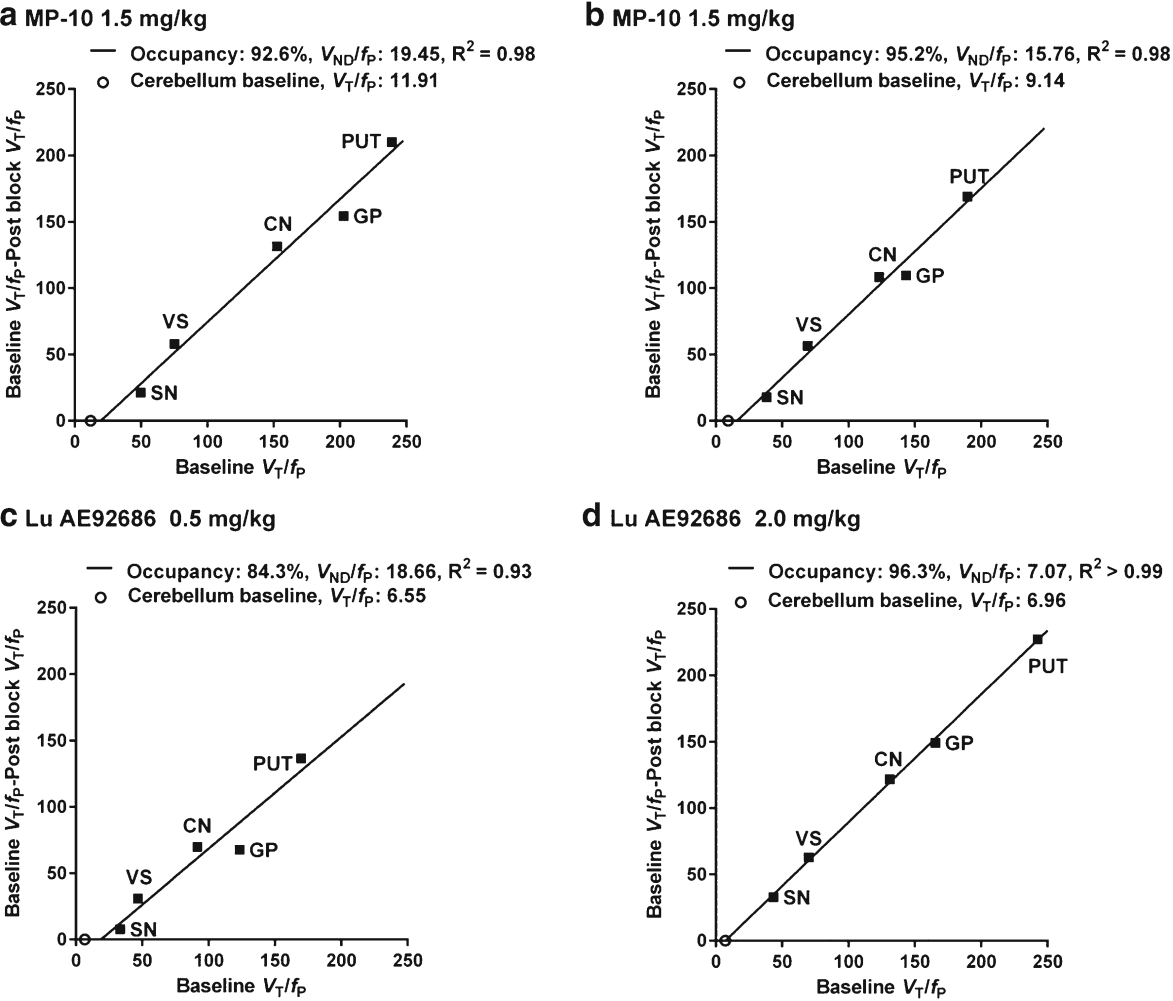
Lassen plots using *V*_T_/*f*_p_ values obtained during baseline conditions and after MP-10 or unlabeled Lu AE92686 pretreatment before injection of [^11^C]Lu AE92686; the regression analysis included five target regions: putamen (PUT), caudate nucleus (CN), globus pallidum (GP), ventral striatum (VS) and substantia nigra (SN) while cerebellum (open circle) was not included in the regression model. (**a**) MP-10 1.5 mg/kg in NHP2 (**b**) MP-10 1.5 mg/kg in NHP1 (**c**) Lu AE92686 0.5 mg/kg (**d**) Lu AE92686 2.0 mg/kg

The PDE10A occupancy after pretreatment was estimated using Lassen plots (Fig. 7). In all studies the goodness of fit (R^2^) for linear fitting was higher than 0.90. In the two NHPs receiving MP-10, the estimated PDE10A occupancy was 93 % and 95 % (Fig. 7a and b). The estimated PDE10A occupancy by Lu AE92686 was 84 % and 96 % after 0.5 mg/kg and 2.0 mg/kg, respectively (Fig. 7c and d). For these four pretreatment experiments, there was a trend towards *V*_ND_/*f*_p_ values (8.6 ± 2.5) being lower than *V*_T_/*f*_p_ values for the cerebellum at baseline conditions (15.2 ± 5.7) (*P* = 0.08).

## Reference tissue models

**Fig. 8:**
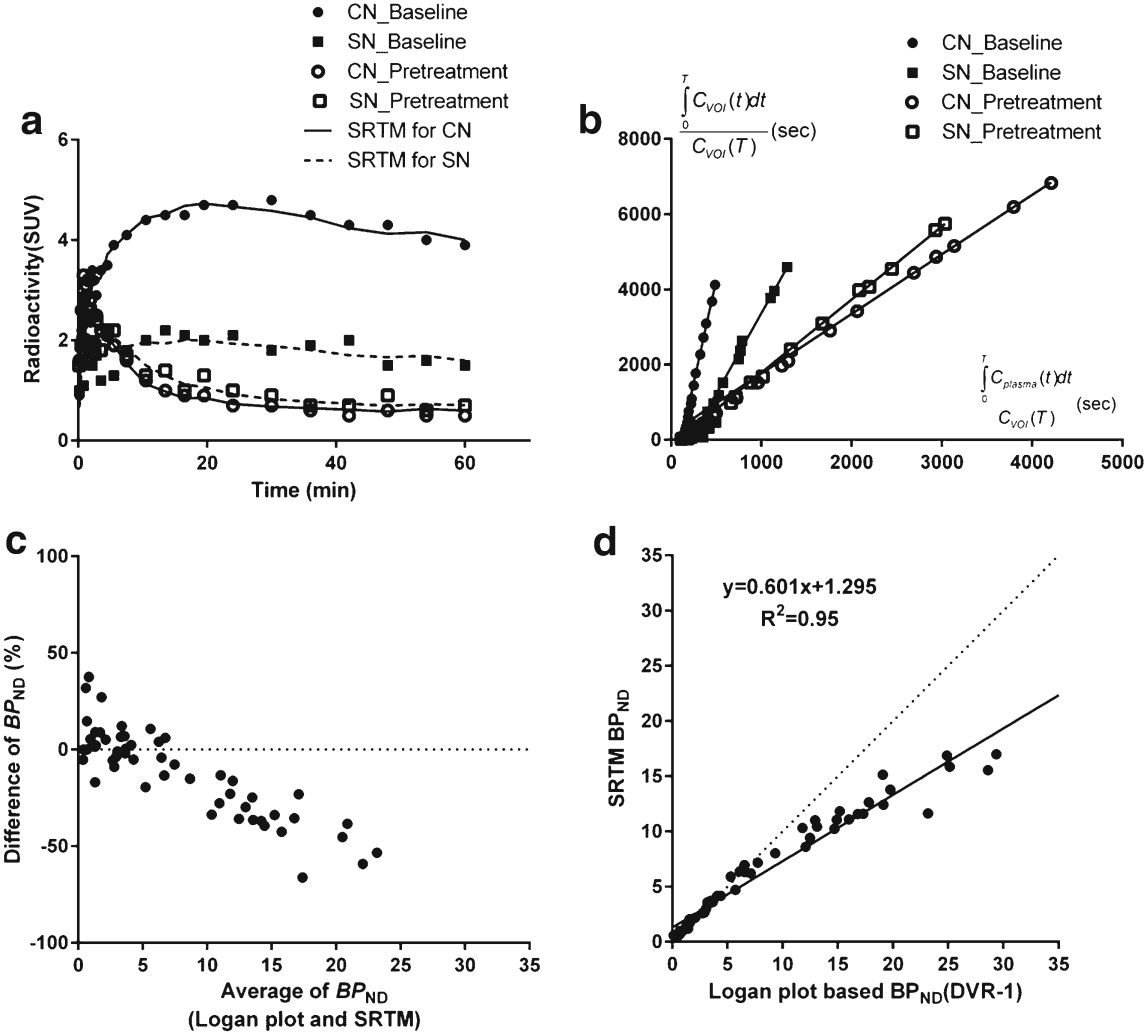
Representative results of simplified reference tissue model (SRTM) and Logan reference tissue model (Loganref) for 63 min data of [^11^C]Lu AE92686 in one NHP (reference region: cerebellum). (**a**) SRTM fits in caudate nucleus (CN) and substantia nigra (SN) for baseline and Lu AE92686 2.0 mg/kg pretreatment condition (**b**) Loganref fits in CN and SN for baseline and Lu AE92686 2.0 mg/kg pretreatment condition (**c**) Bland-Altman plot; Difference of *BP*_ND_ (%) = [(SRTM - Logan plot analysis) / Logan plot analysis] × 100 (**d**) Linear regression analysis; dotted lines represent the line of identity

The cerebellum was used as a reference region, since there was no significant reduction of binding after pretreatment. The baseline *V*_T_ values of target regions and cerebellum are given in Table 3. *BP*_ND_ values could be derived in all experiments by using SRTM or Loganref (Fig. 8a and b). The baseline *BP*_ND_ values calculated by the different models are presented in Table 3. Since the results of the two reference tissue models were comparable (absolute difference: 5.0 ± 4.4 %, Pearson *r* > 0.99 and *P* < 0.0001), only the results of the SRTM are reported in the following sections.

**Table 3:** *V*_T_ values for all baseline experiments (*n* = 7) by Logan plot analysis and comparisons to baseline *BP*_ND_ values estimated by different reference tissue models

	*V*_T_	%COV	*BP*_ND_			% COV
	Logan plots	Logan plots	Logan plots	SRTM	Loganref	SRTM
PUT	12.3 ± 4.5	3.8 ± 2.2	24.3 ± 6.7	15.0 ± 2.1	15.0 ± 2.3	2.2 ± 0.3
CN	7.2 ± 2.5	3.3 ± 2.6	13.8 ± 3.1	10.4 ± 1.3	10.0 ± 1.2	2.1 ± 0.4
GP	8.8 ± 2.5	7.9 ± 1.4	17.2 ± 3.4	11.2 ± 1.4	11.1 ± 1.4	3.6 ± 1.1
VS	3.6 ± 1.2	3.8 ± 2.6	6.5 ± 1.7	6.1 ± 1.1	5.7 ± 1.1	4.3 ± 1.2
SN	2.3 ± 0.5	7.7 ± 2.6	3.8 ± 0.8	3.7 ± 0.4	3.4 ± 0.5	8.9 ± 2.0
CB	0.5 ± 0.1	8.8 ± 1.0	NA	NA	NA	NA

Data presented as mean ± SD

*COV* the coefficient of variation, *PUT* Putamen, *CN* Caudate nucleus, *GP* Globus pallidum, *VS* Ventral striatum, *SN* Substantia nigra, *CB* Cerebellum, *SRTM* simplified reference tissue model, *Loganref* Logan reference tissue model, *NA* not applicable

The *BP*_ND_ values calculated by SRTM in the baseline measurements were lower than those derived indirectly by Logan plot analysis (−19.7 ± 18.2 %, median: −23.3 %) and the difference became mildly positive in pretreatment experiments (5.8 ± 16.8 %, median: 1.5 %) (Fig. 8c). The negative difference (%) became larger at higher *BP*_ND_ values both for baseline measurements (Pearson *r* = −0.88 and *P* < 0.0001) and pretreatment measurements (Pearson *r* = −0.50 and *P* = 0.03). There was a significant correlation between *BP*_ND_ values calculated by Logan plot analysis and SRTM in the baseline measurements (Pearson *r* = 0.95, *P* < 0.0001) and in the pretreatment measurements (Pearson *r* = 0.98, *P* < 0.0001). When pooling baseline and pretreatment data, the equation for the linear regression analysis was y = 0.601x + 1.295 (R^2^ = 0.95) (Fig. 8d).

## Discussion

In the current study, we conducted kinetic analyses of [^11^C]Lu AE92686 to evaluate the suitability of this radioligand for PET examinations of regional PDE10A binding in the monkey brain. There are three main observations: first, regional [^11^C]Lu AE92686 binding could be quantified in the striatum, GP and SN using Logan plot analysis as well as reference tissue models. Second, pretreatment studies confirmed the specificity of [^11^C]Lu AE92686 binding to PDE10A, and there was no evident effect of pretreatment on PDE10A binding in the cerebellum. Third, an influence of blood–brain barrier (BBB) penetrating radiometabolites on brain radioactivity could not be excluded and was minimized by using PET data only for the first 63 min after injection. In conclusion, [^11^C]Lu AE92686 has potential for examinations of PDE10A binding not only in the striatum and GP but also in the SN in future NHP PET studies.

Kinetic analyses of [^11^C]Lu AE92686 suggested that both the 1TCM and 2TCM could describe the TACs for regions with high PDE10A binding. However, the TACs were best described with the 2TCM in regions with low or no specific binding such as SN or cerebellum. The results for the striatum and cerebellum are compatible with a previous evaluation of [^11^C]Lu AE92686 in humans, though the TACs for the human cerebellum were best described by the irreversible 2TCM in the previous study [9], we found no significant difference in MSC values between reversible and irreversible 2TCM. A possible explanation is that some small compartments may represent a larger part of the total signal in regions with low or no specific binding and, thus, are identified by the model.

Overall, the radioactivity in the cerebellum was about 10 % of radioactivity in the putamen (Fig. 3a). The low signal for the cerebellum could be a reason for poor identifiability of *V*_T_ values in this region when using 2TCM [9]. In contrast, Logan plot analysis provided less variable *V*_T_ values in all regions with a %COV < 10 %. Therefore, the Logan plot analysis is the recommended quantification method for estimating *V*_T_ values by [^11^C]Lu AE92686 in NHPs and in humans [9].

In agreement with a previous report in humans [9], there were no major differences between the two examined reference tissue models, SRTM and Loganref, used to quantify regional [^11^C]Lu AE92686 binding. When considering the reference tissue model performance criteria recently proposed by Zanderigo and coworkers [32], it can be noted that the correlation between pooled *BP*_ND_ values of reference tissue models and Logan plot analysis was high (>0.95, proposed: >0.5) and the median percent difference of *BP*_ND_ values between reference tissue models and Logan plot analysis was low (1.5 % to 23.3 %, proposed: <50 %). Fulfillment of these two criteria suggests that SRTM and Loganref may be used to quantify [^11^C]Lu AE92686 binding with acceptable accuracy.

In addition to the two criteria mentioned above, the third proposed performance criterion is a slope of the linear regression line of *BP*_ND_ values between reference tissue models and AIF-based quantification method within the range of 0.7–1.3 (corresponding to a maximum relative difference ≤ 30 %) [32]. In the current study, the slope was 0.6 suggesting that the maximum relative difference in reference tissue models was higher than the proposed level. The deviation between the regression line and the line of identity is mainly driven by regions with high PDE10A density (Fig. 8c and d). Since the current *BP*_ND_ values in target regions are relatively high (>15), a relatively large maximum difference in *BP*_ND_ estimates by SRTM can be anticipated. Similar observations have previously been reported for several other radioligands, including [^11^C]Cimbi-36, [^11^C]WAY-100635 and [^11^C]FLB 457 [24, 33–35]. As for these established radioligands, reference tissue models may be considered for quantification of [^11^C]Lu AE92686 binding in NHP studies in particular when multiple PET measurements are performed on the same subject such as in pharmacological challenge studies [32, 34, 36].

The pretreatment studies in the current study extend reported work, which has been performed in NHP without AIF [9]. The high reduction of [^11^C]Lu AE92686 binding in target regions after pretreatment with MP-10, a PDE10A inhibitor from a different structural class than Lu AE92686, supports the view that [^11^C]Lu AE92686 binds specifically to PDE10A. In all pretreatment studies, the fit of the Lassen plots was good (R^2^ from 0.93 to >0.99). These results suggest that the regional PDE10A occupancy was homogenous and that all five target regions have a similar *V*_ND_ [31].

In addition, pretreatment by MP-10 or Lu AE92686 did not significantly reduce the cerebellar *V*_T_/*f*_p_ values. The discrepancies between estimated *V*_ND_/*f*_p_ values and baseline cerebellum *V*_T_/*f*_p_ values were possibly attributed to the noise in baseline *V*_T_/*f*_p_ values. Such noise violates the assumption of noiseless data in the x-axis of the standard linear regression model in the Lassen plots used for calculation of occupancy [37]. Overall, these results support the use of cerebellum as a reference region in the quantification of [^11^C]Lu AE92686 binding.

The TACs of the cerebellum were better described by the 2TCM than the 1TCM, similarly as has been described for several other PDE10A radioligands [10, 11, 14, 16, 18]. Possible explanations for this include a small fraction of specific binding, a kinetically distinguishable nondisplaceable compartment, tissue heterogeneity (gray and white matter), or a contamination from BBB-penetrating radiometabolites [38, 39]. A presence of BBB-penetrating radiometabolites was supported by the continuous increase in cerebellar *V*_T_ values with increasing PET measurement duration [40]. In contrast, *V*_T_ values in target regions remained stable with increasing PET measurement duration from 63 to 123 min. Therefore, the potential BBB-penetrating radiometabolites mainly influenced the quantification of radioactivity for the cerebellum.

To minimize the potential contribution from radiometabolites, it is preferred to apply the shortest PET measurement duration, which is sufficient for reliable estimation of *V*_T_ values by Logan plot analysis. Using 63 min PET data, reliable *V*_T_ values could be derived by Logan plot analysis both in target regions and cerebellum. PET measurement duration shorter than 63 min resulted in variable and poor identifiable *V*_T_ estimates in several regions. Furthermore, it should be considered that due to the rapid decay of ^11^C (half-life = 20 min), a longer measurement duration might introduce more noise both in the image and blood data. Shortening of the PET measurement duration has also been proposed for the application of other PDE10A radioligand, e.g. [^18^F]JNJ-42259152 in rats, to overcome the confounding effects from BBB-penetrating radiometabolites [17]. Therefore, 63 min is suggested as the preferred PET measurement duration for [^11^C]Lu AE92686 binding in cynomolgus monkeys.

The metabolic rate of [^11^C]Lu AE92686 in monkeys is similar to rats, but more rapid than previously reported in humans [9]. It is worth noting that we compared the fraction of unchanged [^11^C]Lu AE92686 to the total amount of radioactivity in plasma across species. This relative fraction of unchanged [^11^C]Lu AE92686 in plasma is dependent on the clearance rate of the radiometabolites in plasma, a rate which also may differ across species. However, in general, the potential difference in metabolic rate across species is consistent with less extensive metabolism in higher species, an observation previously reported for other radioligands [10, 11, 16, 17, 41].

The radiometabolites observed early in monkey plasma were more hydrophilic than [^11^C]Lu AE92686, similar as in rat and human [9]. These observations are consistent with the results of a metabolic soft-spot mass spectrometry analysis of Lu AE92686. After incubation in rat and human liver microsomes, only hydroxy and di-hydroxy metabolites were suggested, and all metabolites were more hydrophilic than the parent compound (unpublished data, data on file at Lundbeck). It has been reported that at 40 min post-injection, the amount of radiometabolites in rat brain is small (<15 %) [9]. It may thus be anticipated that the majority of radioactivity in the NHP brain at 60 min post-injection would be [^11^C]Lu AE92686. Ideally, the structure of the metabolites would be identified and, if possible, the metabolites radiolabeled to examine their passage across the BBB. Further studies are warranted to characterize these radiometabolites and to further confirm their influence on the quantification of [^11^C]Lu AE92686 in the NHP brain.

A large number of PET radioligands have been developed for the PDE10A enzyme [8–19]. Table 4 summarizes the characteristics of PDE10A radioligands that have been applied in primates. After adjustment for the differences in target VOI (putamen vs. striatum) and PET measurement duration (63 min vs. 90 min), the striatal *BP*_ND_ values (12.0 ± 1.6) in the current study were 85 % higher than previously reported values with [^11^C]Lu AE92686 in cynomolgus monkeys [9]. This difference may be caused by an enhanced striatum signal in the current study due to the resolution of the PET systems (1.6 mm vs. 3.5 mm) and to the use of a head fixation device, limiting head motion in the current study. Similar differences between PET systems have been reported in imaging studies of dopamine or serotonin transporters, with an increase in *BP*_ND_ values of up to 92 % when using the HRRT [42, 43]. It is noted that the majority of the studies of PDE10A radioligands did not in detail evaluate the influence of radiometabolites on quantification. For example, only studies using [^18^F]JNJ-42259152 [16] or [^18^F]MNI-659 [18] have examined the effect of PET measurement duration on the quantification, and for both radioligands an increase in *V*_T_ value was obtained with increasing PET measurement duration.

**Table 4:** Summary of PDE10A PET radioligands evaluated in primates^a^

Radioligand	Species	PreTx	Duration effect^c^	Reference region	PUT	SN	TRT (%)	Ref
[^11^C]Lu AE92686	Human (*n* = 6)	NA	NA	CB	7.5^d^	NA	6	9
	Cynomolgus (*n* = 2)	A^b^	NA	CB	6.5^d^	NA	NA	9
	Cynomolgus (*n* = 5)	A	A^b^	CB	15.0	3.7	NA	*
[^11^C]MP-10	Baboon (*n* = 4)	A	NA	CB	1.6	NA	NA	10
	Rhesus (*n* = 4)	A	NA	NA	NA	NA	NA	11
[^11^C]IMA107	Baboon (*n* = 2)	A	NA	CB	4.3	0.5^e^	NA	08/12
[^11^C]AMG 7980	Baboon (*n* = 2)	A	NA	Thalamus	0.8	NA	NA	13
[^11^C]T-773	Rhesus (*n* = 2)	A	NA	CB	2.2	NA	NA	14
[^11^C]TZ1964B	Cynomolgus (*n* = 2)	A^b^	NA	CB	4.6^d^	NA	NA	15
[^18^F]JNJ-42259152	Human (*n* = 12)	NA	A	Frontal ctx	3.5	0.4	5–12	16
[^18^F]MNI-659	Human (*n* = 5)	NA	A	CB	3.8	NA	5–8	18
[^18^F]AMG 580	Rhesus (*n* = 1)	NA	NA	Midbrain	2.9	NA	NA	19
	Baboon (*n* = 2)	NA	NA	Thalamus	NA	NA	NA	19

*PreTx* pretreatment experiment with PDE10A inhibitor or unlabelled radioligand, *NA* not applicable, *A* available, *CB* Cerebellum, *Frontal ctx* Frontal cortex, *PUT BP*_ND_ values in putamen, *SN BP*_ND_ values in substantia nigra, *TRT (%)* test-retest variability (%) for *BP*_ND_ values = $\left[\frac{\left|\mathrm{test}-\mathrm{retest}\right|}{\left(\frac{\mathrm{test}+\mathrm{retest}}{2}\right)}\right]\times 100$

^a^Based on the literature describing the quantification or characterization of the radioligands

^b^No arterial input function

^c^Effect of PET measurement duration on quantification

^d^ *BP*_ND_ values in striatum

^e^Reported in a human study (*n* = 12, healthy subjects)

*Current study

[^11^C]Lu AE92686 binding in SN could be quantified with favorable signal to noise ratio. The *BP*_ND_ for SN was 3–5, which is higher than ∼0.5 as reported in studies using other PDE10A radioligands and lower resolution PET systems [8, 16] (Table 4). A head-to-head comparison on the HRRT is required to allow for a direct comparison of SN binding by different PDE10A radioligands. In general, considering the relative small size of SN (56 ± 14 mm^3^ in the current study), *BP*_ND_ values higher than 0.5 have been suggested to be required for reliable quantification [20]. The pretreatment studies in the current study confirm that the nondisplaceable binding in the SN is similar to that in the striatum. In conclusion, [^11^C]Lu AE92686 may allow for the unique potential to quantify PDE10A binding in the SN. Such studies in nonhuman primates and humans may lead to a better understanding of the role of PDE10A in the pathophysiology and treatment of neuropsychiatric diseases.

A limitation of the current study is the use of anesthesia during PET measurements. The induction by ketamine and maintenance by sevoflurane is similar to the previously reported monkey study with [^11^C]Lu AE92686 [9]. The influence of these anesthetics on the expression of PDE10A or cAMP concentration is to our knowledge not known. However, the striatal binding of [^11^C]Lu AE92686 in awake humans and anaesthetized monkeys has been reported to be comparable in a previous study [9]. Therefore, the anesthesia effects on the binding of [^11^C]Lu AE92686 are unlikely to be prominent.

In conclusion, our results suggest that [^11^C]Lu AE92686 can be applied to examine PDE10A binding in striatum, GP and SN. A potential contribution from radiometabolites can be minimized by shortening the PET measurement duration to 63 min. Reliable *BP*_ND_ estimates could be derived by reference tissue models (SRTM and Loganref) and these models are the preferred methods in future pharmacological challenge studies in NHPs.

## Electronic supplementary material

Below is the link to the electronic supplementary material.

ESM 1 (PDF 304 kb)

ESM 2 (PDF 255 kb)
