# Supplementary material for: Characterization of [11C]Lu AE92686 as a PET radioligand for phosphodiesterase 10A in the nonhuman primate brain
Source: Eur J Nucl Med Mol Imaging. 2016 Nov 5;44(2):308–20. doi: 10.1007/s00259-016-3544-9 (PMC5215309; doi:10.1007/s00259-016-3544-9)
Supplement: Supplementary file 1 — (PDF 304 kb) [file 259_2016_3544_MOESM1_ESM.pdf]

EUR J NUCL MED MOLEC IMAGING

Characterization of [^{11}C]Lu AE92686 as a PET radioligand for phosphodiesterase 10A in the nonhuman primate brain

Authors: Kai-Chun Yang[1], Vladimir Stepanov[1], Nahid Amini[1], Stefan Martinsson[1], Akihiro Takano[1], Jacob Nielsen[2], Christoffer Bundgaard[3], Benny Bang-Andersen[3], Sarah Grimwood[4], Christer Halldin[1], Lars Farde[1, 5], Sjoerd J. Finnema[1, #]

Affiliations: [1] Department of Clinical Neuroscience, Center for Psychiatric Research, Karolinska Institutet, Karolinska University Hospital, Stockholm, Sweden; [2] Synaptic Transmission, H. Lundbeck A/S, Valby, Denmark; [3] Discovery Chemistry and DMPK, H. Lundbeck A/S, Valby, Denmark; [4] Neuroscience and Pain Research Unit, Pfizer Inc., Cambridge, MA, USA; [5] Personalized Health Care and Biomarkers, AstraZeneca PET Science Center at Karolinska Institutet, Stockholm, Sweden

[#]current affilliation: Yale University, Department of Radiology and Biomedical Imaging, New Haven, CT, USA

Address of Corresponding author: Kai-Chun Yang, Department of Clinical Neuroscience, Center for Psychiatric Research, Karolinska Institutet, Karolinska University Hospital, Building R5:02, SE-17176 Stockholm, Sweden. Tel. Nr: +46-8-51772997, Fax. Nr: +46-8-51771753, Email: kai-chun.yang@ki.se

SUPPLEMENTAL METHODS

Preparation of [^{11}C]Lu AE92686

[^{11}C]Lu AE92686 (5,8-dimethyl-2-(2-(1-[^{11}C]methyl-4-phenyl-1H-imidazol-2-yl)ethyl)-[1,2,4]triazolo[1,5-a]pyridine) was synthesized from its desmethylated precursor (5,8-dimethyl-2-(2-(4-phenyl-1H-imidazol-2-yl)ethyl)-[1,2,4]triazolo[1,5-a]pyridine) by reacting the precursor with [^{11}C]methyl iodide, with DMF as a reaction solvent (0.4-0.5 ml) and solid NaOH (1.5-2.0 mg) as base. The reaction vessel was heated to 70 ^{o}C for 3 minutes. After heating, the reaction mixture was diluted with water and injected onto a Waters XBridge C18 (250×10 mm) HPLC column and the column was eluted with mobile phase consisting of 350:650 mixture of acetonitrile/aq. triethylamine (0.1%) at a flow of 5 ml/min. The product fraction was collected into 60 ml of 0.1% sodium ascorbate solution and concentrated on a Oasis HLB 1cc SPE cartridge. The cartridge was then washed with sterile water and the product eluted with ca. 1.0 ml of 99.6% ethanol into a vial containing ca. 12 ml of phosphate buffered saline. The final formulation was filtered though sterile Millipore Millex GV 0.22 µm filter. The QC sample was then taken from the product vial and radiochemical purity assessed by co-injecting it with an unlabeled reference using Waters XBridge C18 (150×4.6 mm) column, eluted with acetonitrile/aq. phosphoric acid (0.05M) 270:730 at a flow of 2 ml/min, with UV detector set to 220 nm. An average production batch resulted in 1.3-2.2 GBq of [^{11}C]Lu AE92686. Average synthesis time was ca. 35 min.

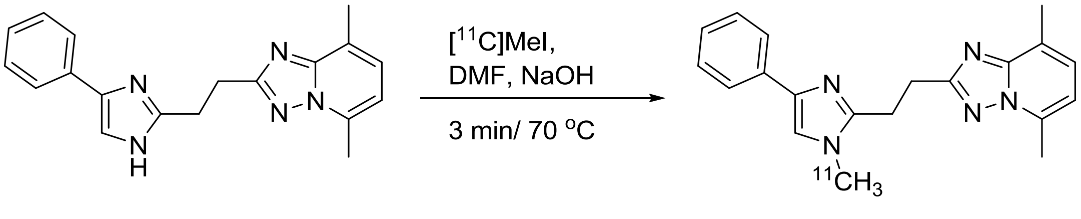

Fig. 1 Preparation of [^{11}C]Lu AE92686

Magnetic resonance imaging (MRI) measurements

Each subject underwent a MRI measurement to control for intracranial brain lesions and for anatomical guidance in the definition of the volumes of interest (VOIs). T1-weighted MRI images were acquired on a GE 1.5 Tesla Signa MRI scanner (Milwaukee, WI) with a 3D spoiled gradient recalled (SPGR) protocol: repetition time 21 ms, flip angle 35°, FOV 12.8, matrix 256 × 256 × 128, 128 × 1.0 mm^2 slices.

Measurement of protein binding

The same ultrafiltration method applied in previous studies [1, 2] was used to estimate the free fraction (f_p) of [^{11}C]Lu AE92686 in plasma. Monkey plasma (500 µL) or a phosphate buffered saline (PBS) solution (pH 7.4) (500 µL) as a control, was mixed with a [^{11}C]Lu AE92686 solution (50 µL, ~1 MBq) and incubated at room temperature for 10 min. After the incubation, 200 µL portions of the incubation mixtures were pipetted into ultra-filtration tubes (Centrifree YM-30; Millipore, Billerica, MA, USA) and centrifuged at 1500*g* for 15 min. Equal aliquots (20 µL) of the ultrafiltrate (C_{free}) and of the plasma (C_{total}) were counted with a NaI well-counter. Each determination was performed in duplicate. The free fraction of [^{11}C]Lu AE92686 was calculated as $f_p = C_{free} / C_{total}$ and the results were corrected for membrane binding measured by the control samples.

Radiometabolite analysis

A reversed-phase high-performance liquid chromatography (HPLC) method was performed to determine the percentages of radioactivity from unchanged radioligand and radiometabolites in monkey plasma. The plasma obtained after centrifugation of blood at 2000*g* for 2 min was mixed with 1.4 times volume of acetonitrile. After stirring with a vortex mixer, the sample was centrifuged at 2000 *g* for 4 min and 2–3 mL of water was added to the

supernatant plasma-acetonitrile mixture, which was then injected to a radio-HPLC system. The blood (1.0–3.0 mL) and plasma (0.5–1.5 mL) were counted in a NaI well-counter. The radioactivity of protein precipitate fraction was also measured to quantify the recovery after precipitation with acetonitrile. The radio-HPLC system used in the plasma experiments consisted of an interface module (D-7000; Hitachi), a pump (L-7100; Hitachi), an injector (7125, 5.0 mL loop; Rheodyne, Cotati, CA, USA), an UV absorbance detector (L-7400, λ = 280 nm; Hitachi) in series with a 150TR; Packard radioactivity detector (housed in a shield of 50 mm thick lead and equipped with a 550 mL flow cell). Chromatographic separation was achieved on a XBridge C18 column, (50 mm × 10 mm I.D., 2.5 µm + 10 mm × 10 mm I.D., 5 µm; Waters, USA) by gradient elution. Acetonitrile (A) and 20 mM ammonium phosphate (pH 7) (B) were used as the mobile phase at 6.0 mL/min, according to the following program: 0–3.5 min (A/B), 20/80 → 75/25 v/v; 3.5–4.0 min (A/B), 75/25 v/v; and 4.0–4.1 min (A/B), 75/25 → 20/80 v/v.

Determination of plasma drug concentrations

Plasma samples were analysed at H. Lundbeck A/S using a Waters Acquity ultra performance liquid chromatography (UPLC) system (Waters, Milford, MA, USA) followed by tandem mass spectrometry (MS/MS) detection with a Sciex API4000 (AB Sciex, Foster City, CA, USA). Individual standard curves were established for each analyte from the MS peak areas which correlated linearly with the plasma concentration of the analytes in the range of 1.0–1000 ng/ml.

SUPPLEMENTAL REFERENCES

1. Takano A, Stepanov V, Gulyás B, Nakao R, Amini N, Miura S et al. Evaluation of a novel PDE10A PET radioligand, [^{11}C]T-773, in nonhuman primates: Brain and whole body PET and brain autoradiography. Synapse. 2015;69(7):345-55.

2. Finnema SJ, Stepanov V, Ettrup A, Nakao R, Amini N, Svedberg M et al. Characterization of [^{11}C]Cimbi-36 as an agonist PET radioligand for the 5-HT_{2A} and 5-HT_{2C} receptors in the nonhuman primate brain. Neuroimage. 2014;84:342-53.
