# Supplementary material for: Characterization of [11C]Lu AE92686 as a PET radioligand for phosphodiesterase 10A in the nonhuman primate brain
Source: Eur J Nucl Med Mol Imaging. 2016 Nov 5;44(2):308–20. doi: 10.1007/s00259-016-3544-9 (PMC5215309; doi:10.1007/s00259-016-3544-9)
Supplement: Supplementary file 2 — (PDF 255 kb) [file 259_2016_3544_MOESM2_ESM.pdf]

EUR J NUCL MED MOLEC IMAGING

Characterization of [^{11}C]Lu AE92686 as a PET radioligand for phosphodiesterase 10A in the nonhuman primate brain

Authors: Kai-Chun Yang[1], Vladimir Stepanov[1], Nahid Amini[1], Stefan Martinsson[1], Akihiro Takano[1], Jacob Nielsen[2], Christoffer Bundgaard[3], Benny Bang-Andersen[3], Sarah Grimwood[4], Christer Halldin[1], Lars Farde[1, 5], Sjoerd J. Finnema[1, #]

Affiliations: [1] Department of Clinical Neuroscience, Center for Psychiatric Research, Karolinska Institutet, Karolinska University Hospital, Stockholm, Sweden; [2] Synaptic Transmission, H. Lundbeck A/S, Valby, Denmark; [3] Discovery Chemistry and DMPK, H. Lundbeck A/S, Valby, Denmark; [4] Neuroscience and Pain Research Unit, Pfizer Inc., Cambridge, MA, USA; [5] Personalized Health Care and Biomarkers, AstraZeneca PET Science Center at Karolinska Institutet, Stockholm, Sweden

[#]current affilliation: Yale University, Department of Radiology and Biomedical Imaging, New Haven, CT, USA

Address of Corresponding author: Kai-Chun Yang, Department of Clinical Neuroscience, Center for Psychiatric Research, Karolinska Institutet, Karolinska University Hospital, Building R5:02, SE-17176 Stockholm, Sweden. Tel. Nr: +46-8-51772997, Fax. Nr: +46-8-51771753, Email: kai-chun.yang@ki.se

SUPPLEMENTAL RESULTS

FIGURE LEGENDS

Fig. S1 Plasma concentration of pretreatment drugs in cynomolgus monkeys. (a) MP-10 1.5mg/kg in NHP1 (b) MP-10 1.5mg/kg in NHP2 (c) Lu AE92686 0.5 mg/kg (d) Lu AE92686 2.0 mg/kg

Fig. S2 Plasma fraction of unchanged [^{11}C]Lu AE92686 over time for baseline and pretreatment experiments. (a) MP-10 1.5mg/kg in NHP1 (b) MP-10 1.5mg/kg in NHP2 (c) Lu AE92686 0.5 mg/kg (d) Lu AE92686 2.0 mg/kg

Fig. S1

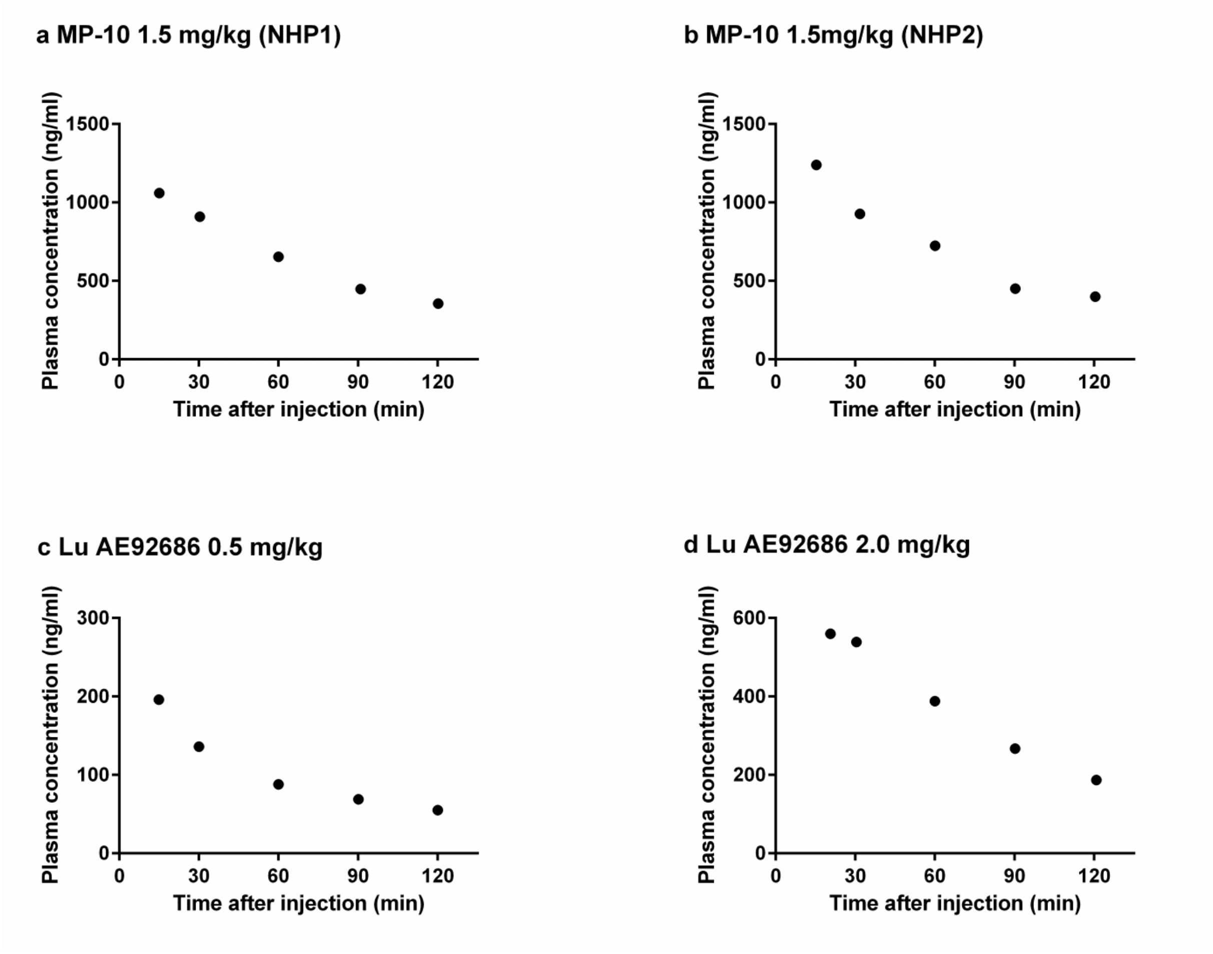
a MP-10 1.5 mg/kg (NHP1)
Plasma concentration (ng/ml)
1500
1000
500
0
0
30
60
90
120
Time after injection (min)
b MP-10 1.5mg/kg (NHP2)
Plasma concentration (ng/ml)
1500
1000
500
0
0
30
60
90
120
Time after injection (min)
c Lu AE92686 0.5 mg/kg
Plasma concentration (ng/ml)
300
200
100
0
0
30
60
90
120
Time after injection (min)
d Lu AE92686 2.0 mg/kg
Plasma concentration (ng/ml)
600
400
200
0
0
30
60
90
120
Time after injection (min)

Fig. S2

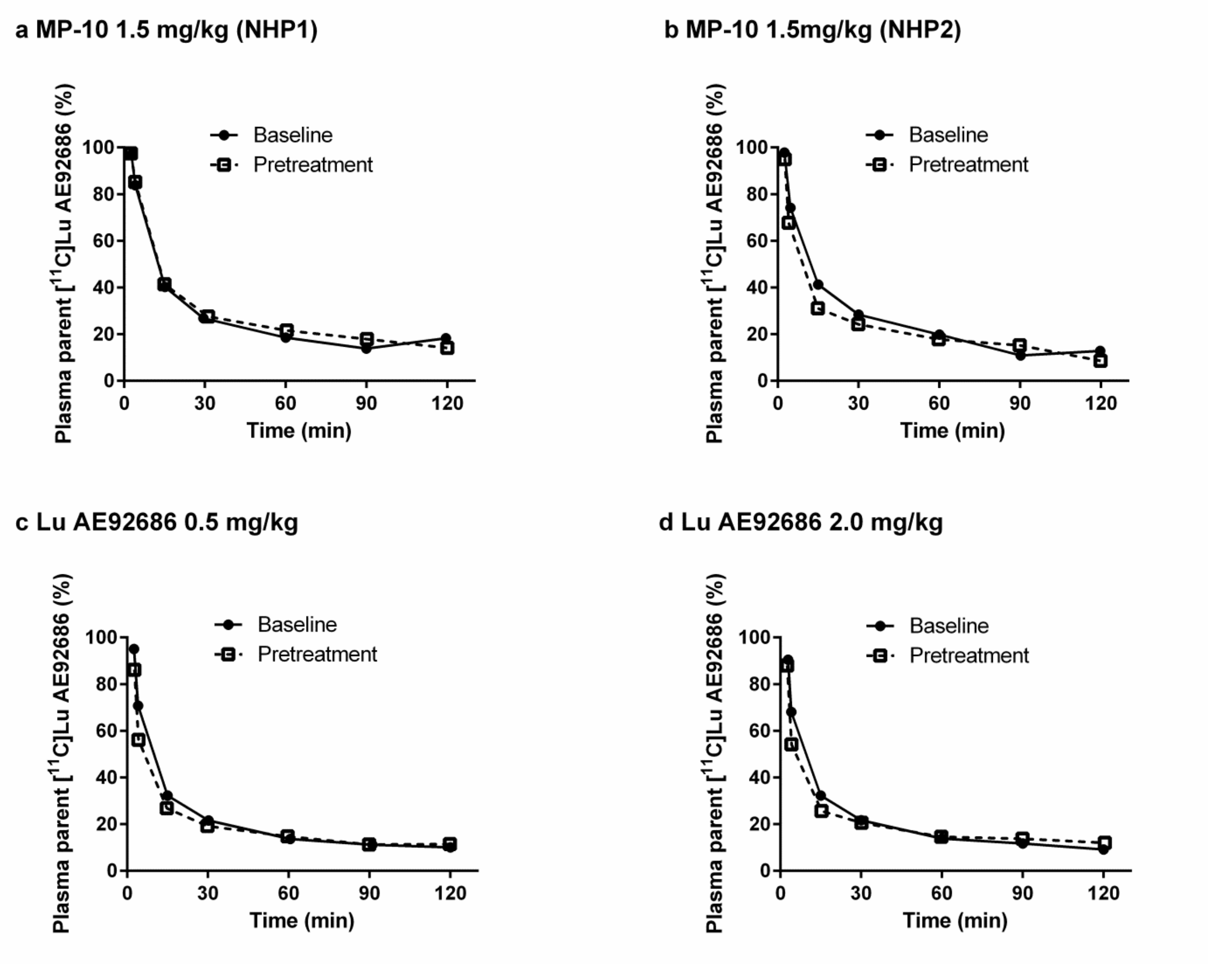
a MP-10 1.5 mg/kg (NHP1)
b MP-10 1.5mg/kg (NHP2)
c Lu AE92686 0.5 mg/kg
d Lu AE92686 2.0 mg/kg
Baseline
Pretreatment
Plasma parent [11C]Lu AE92686 (%)
Time (min)
0
20
40
60
80
100
30
60
90
120
